# *Porphyromonas gingivalis* induces an inflammatory response via the cGAS-STING signaling pathway in a periodontitis mouse model

**DOI:** 10.3389/fmicb.2023.1183415

**Published:** 2023-06-19

**Authors:** Rong Bi, Yanling Yang, Hongwei Liao, Guang Ji, Yan Ma, Lukui Cai, Jingyan Li, Jingsi Yang, Mingbo Sun, Jiangli Liang, Li Shi

**Affiliations:** ^1^Laboratory of Immunogenetics, Institute of Medical Biology, Chinese Academy of Medical Science, Peking Union Medical College, Kunming, Yunnan, China; ^2^The Affiliated Stomatology Hospital of Kunming Medical University, Center of Stomatology, Affiliated Hospital of Yunnan University, Kunming, Yunnan, China; ^3^Laboratory of Vaccine Development, Institute of Medical Biology, Chinese Academy of Medical Sciences, Peking Union Medical College, Kunming, China

**Keywords:** periodontal disease, *Porphyromonas gingivalis*, cGAS-STING signaling pathway, proinflammatory cytokines, RANKL, osteoclast, macrophage polarization

## Abstract

Periodontitis is an inflammatory disease initiated by periodontopathogenic bacteria in the dental plaque biofilms. Understanding the role of *Porphyromonas gingivalis* (*P. gingivalis*), a keystone pathogen associated with chronic periodontitis, in the inflammatory response is crucial. Herein, we investigated whether *P. gingivalis* infection triggers the expression of the type I IFN gene and various cytokines and leads to activation of the cGAMP synthase–stimulator of IFN genes (cGAS-STING) pathway both *in vitro* and in a mouse model. Additionally, in an experimental model of periodontitis using *P. gingivalis*, Sting^Gt^ mice showed lower levels of inflammatory cytokines and bone resorption than wild-type mice. Furthermore, we report that a STING inhibitor (SN-011) significantly decreased inflammatory cytokine production and osteoclast formation in a periodontitis mouse model with *P. gingivalis*. In addition, STING agonist (SR-717) -treated periodontitis mice displayed enhanced macrophage infiltration and M1 macrophage polarization in periodontal lesions compared with that in vehicle-treated periodontitis mice. In conclusion, our results demonstrate that the cGAS-STING signaling pathway may be one of the key mechanisms crucial for the *P. gingivalis*-induced inflammatory response that leads to chronic periodontitis.

## Introduction

Periodontitis is a chronic inflammatory disease in which a polymicrobial infection of anaerobic gram-negative bacteria in the subgingiva triggers a deregulated host immune response that leads to the breakdown of the periodontal ligament and the surrounding alveolar bone (Hajishengallis, 2022). In particular, inflammation persists in periodontal tissues, leading to alveolar bone loss around the teeth (Belibasakis and Bostanci, 2012; Kononen et al., 2019). In 2019, there were 1.1 billion prevalent cases of severe periodontitis globally (Chen et al., 2021). In addition, periodontitis is associated with systemic inflammatory diseases, including diabetes mellitus, Alzheimer’s disease, and cardiovascular disease; furthermore, periodontitis also worsens atherosclerosis (Liccardo et al., 2019). Therefore, periodontitis is becoming a major health concern worldwide, and it is important to understand its pathogenesis in order to establish new treatment methods.

Recent studies of the pathogenesis of periodontitis have revealed that disruption of the homeostasis between the resident microbiota and the host immune response is the major cause of periodontitis (Hajishengallis et al., 2020; Chang et al., 2021). As the keystone periodontitis pathogen, *Porphyromonas gingivalis* (*P. gingivalis*) is capable of remodeling the commensal bacterial community to promote dysbiosis, thus playing a central role during the progression of periodontitis (Nakayama and Ohara, 2017). This pathogen accumulates in subgingival plaque biofilms and invades periodontal tissue, releasing a variety of virulence factors, including cysteine proteases (e.g., gingipains), hemagglutinins, lipopolysaccharides (LPS), fimbriae, and outer membrane vesicles (OMVs; Wada and Kamisaki, 2010; Jia et al., 2019). These virulence factors can cause tissue destruction on their own or act through other mediators to induce inflammation. For example, gingipains [arginine gingipain (RgpA) and lysine gingipain (Kgp)] are important for the invasion of tissue structures, and *P. gingivalis* gingipain, interferes with the cell-mediated immune response and stimulates the expression of protease-activated receptors on gingival epithelial cells by releasing proinflammatory cytokines (Morozumi et al., 2018). The invasion of periodontal tissue by inflammatory cells and the inflammatory response are major factors in the development of periodontitis (Hajishengallis and Lamont, 2021). Recent studies have demonstrated that *P. gingivalis* OMVs activate the extracellular signal-regulated kinase (Erk) 1/2, c-Jun N-terminal kinase (JNK), and NF-κB signaling pathways, resulting in increased IL-6 and IL-8 expression in human gingival epithelial cells (Babamale and Chen, 2021). Numerous studies have also suggested that *P. gingivalis* LPS contributes to the activation of macrophages and the release of inflammatory factors via the TLR signaling pathway (Le Sage et al., 2017). Therefore, regulation of the immune response activated by *P. gingivalis* is crucial for preventing dense immune cell infiltration that can result in chronic inflammatory lesions.

Recent evidence has noted that Type I IFNs play an important role in antimicrobial and antiviral immunity (Liu et al., 2021). Host cells recognize cytosolic double-stranded DNAs (dsDNA) and cyclic dinucleotides (CDN) of bacteria via the innate immune receptor stimulator interferon genes (STING), leading to the production of IFNs (Kato et al., 2017). In the oral cavity, microbes constantly invade oral tissues, extracellular DNA is released from microbial biofilms and damaged host cells, leading to an increase in extracellular DNA (Elmanfi et al., 2021). Various pattern recognition receptors [TLRs, RIG-I-like receptors (RLRs)] may cooperatively work to protect the host against microbial invasion (Nandakumar et al., 2019). When dsDNA binds to cGAS in the cytoplasm, cGAS undergoes a conformational change. In the later phase, ATP and GTP convert the bound cGAS-DNA to 2′3’-cGAMP, an endogenous secondary messenger. Then, the 2′3’-cGAMP isomer serves as a secondary messenger by binding and activating the STING protein. STING can activate protein kinases, TANK-binding kinase 1 (TBK1), IFN regulatory factor 3 (IRF3), resulting in transcription factor release. In addition, these transcription factors translocate into the nucleus and provide a synergistic response against invading pathogens. This translocation stimulates the transcription and expression of genes encoding type I IFNs (such as IFN-β) and various cytokines and chemokines (such as IL-6 and TNF-α; Le Sage et al., 2017). In periodontitis, immunohistochemical analyses showed strong STING accumulation in the basal epithelium and around vessel walls in the connective tissue; however, STING was weakly present in healthy gingiva (Elmanfi et al., 2021). Type I IFNs and the cGAS-STING pathway may play a crucial role in *P. gingivalis* infection. As a result, it is important to further explore the role of the STING pathway in periodontitis pathogenesis.

When periodontal cellular responses (e.g., keratinocytes, fibroblasts, and mononuclear/macrophages) are activated by CDNs, the immune response may be mediated by the secretion of cytokines, but excessive amounts of cytokines may destroy the periodontal tissue and aggravate the progression of periodontal disease (Zhou et al., 2021; Hu et al., 2022). In periodontitis, receptor activator of nuclear factor-κB ligand (RANKL)-producing cells and osteoblasts are activated and differentiated by inflammatory cytokines, leading to osteoclastic differentiation and activation (Usui et al., 2021). In addition to this mechanism, pathogenic periodontal bacteria and mechanical stress also contribute to the activation of osteoclasts and the destruction of alveolar bone (Huang et al., 2020). It has also been reported that mice lacking the RANKL gene or patients with RANKL gene mutations exhibited osteopetrosis with the absence of normal osteoclasts on the surface of the bone (Feng et al., 2019). Therefore, upon stimulation by periodontal bacteria, periodontal ligament cells produce proinflammatory cytokines and modulate osteoclast formation. Recently, Uemura et al. (2022) reported that *Pg*-OMV induces inflammatory cytokines in human gingival epithelial cells by stimulating various pathways. However, there is limited knowledge on whether *P. gingivalis* infection would stimulate inflammatory responses through the cGAS-STING pathway in a periodontitis mouse model, what mechanisms may be involved, and what the functional impact is. As the “gatekeeper” of the immune system, the cGAS-STING pathway plays a critical role in protecting the host from invading bacteria, viruses and other microorganisms (Kato et al., 2017). Thus, it is pivotal to explore the molecular mechanisms by which *P. gingivalis* and the cGAS-STING signaling pathway control the pathogenesis of chronic inflammatory disease.

In this study, we first determined whether proinflammatory cytokines can be produced in response to *P. gingivalis* via the cGAS-STING pathway *in vitro*. We then explored the function of the cGAS-STING signaling pathway in a mouse model of periodontitis. Both wild-type and STING-deficient mouse models of periodontitis were developed, and the activation of the pathway and its downstream inflammatory cytokines were examined. Moreover, we explored the role of STING modulators in *P. gingivalis*-induced proinflammatory cytokine changes in mice with periodontitis and their regulation in relation to STING signaling. In summary, we explored whether *P. gingivalis* contributes to the pathogenesis of the immune response and inflammation by promoting the activation of the cGAS-STING signaling pathway.

## Materials and methods

### Mice and ethnic statement

Five-to 6-week-old male STING knockout, Sting^Gt^ mice generated with the C57BL/6 background (see Supplementary material 2.1), and wild-type (WT) C57BL/6 mice were purchased from The Jackson Laboratory (Nanjing, CHN) and the Vital River Laboratory (Beijing, CHN). All mice used in this study were treated in accordance with the Guide for the Care and Use of Laboratory Animals of the People’s Republic of China. All methods were reviewed and approved by the Committee on Ethics of the Institute of Medical Biology, Chinese Academy of Medical Sciences (IMBCAMS; assurance number: DWSP201912001). The mice were raised and kept at IMBCAMS under SPF conditions with constant temperature (20–24°C), humidity (45–65%), and a light/dark cycle (12 h/12 h).

### Bacterial strain culture

*Porphyromonas gingivalis* BNCC357750 was purchased from BeNa Culture Collection (BNCC, China). *P. gingivalis* was grown for 8–10 days in Columbia blood-agar (CB-A, 5% defibrinated sheep blood, 1 μg/mL hemin, and 1 μg/mL vitamin K1) at 35°C in an anaerobic chamber equilibrated with a mixture of 90% nitrogen, 5% carbon dioxide, and 5% hydrogen. Colonies were picked from fresh CB-A plates and resuspended in phosphate-buffered saline (PBS; Solarbio, CHN), diluted to a concentration of 5 × 10^10^ CFU/mL by a turbidimetric technique, and then used within 2 h.

### Ligature ligation and *Porphyromonas gingivalis*–induced periodontitis mouse model

**Figure 1:**
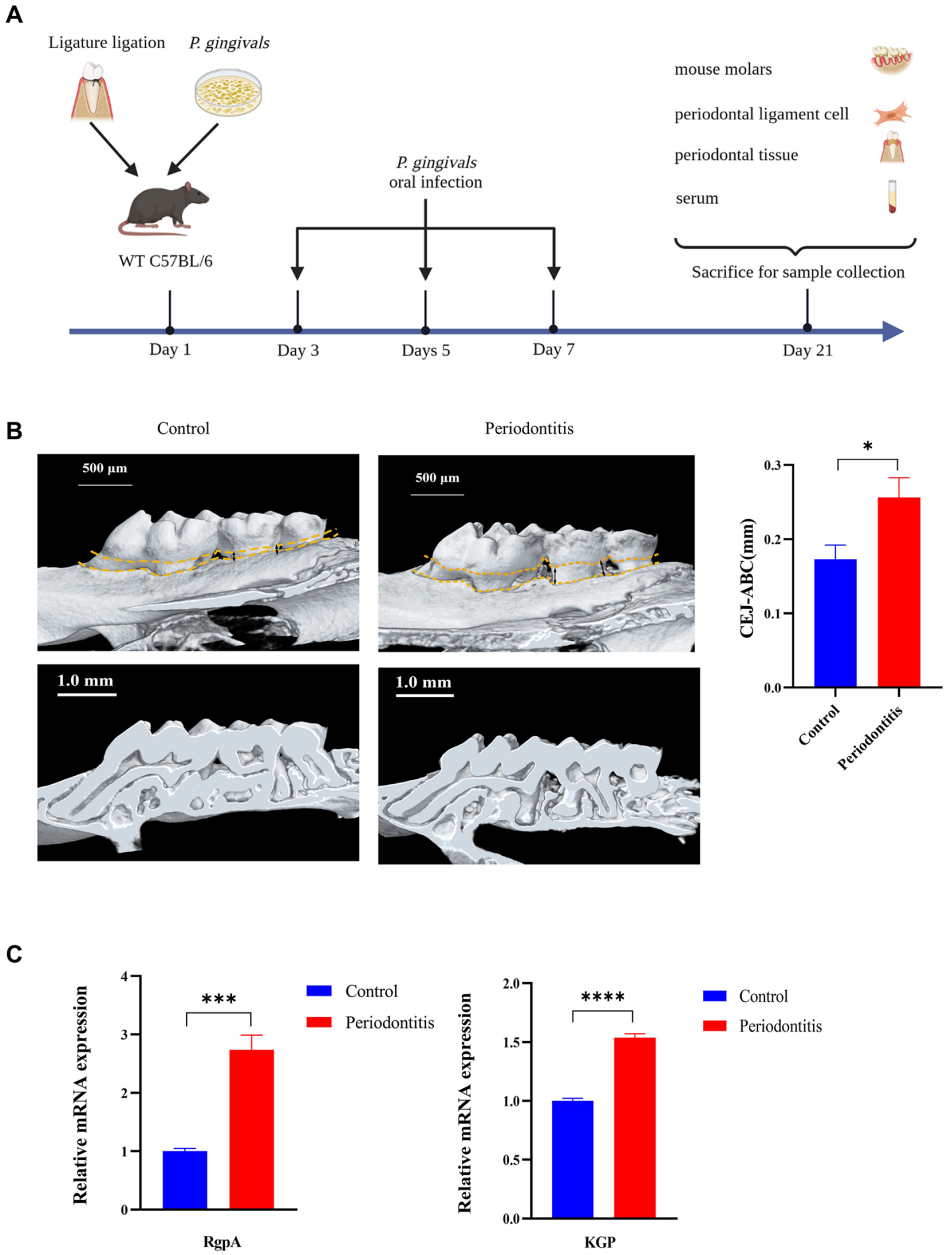
Successful construction of a *Porphyromonas gingivalis*-infected animal model of periodontitis. C57BL/6 mice were used to establish the periodontitis model, and uninfected mice served as the control group (*n* = 4 mice per group). **(A)** Schematic of the mouse periodontitis model. **(B)** Three-dimensional reconstruction images and analysis of the cementoenamel junction to alveolar bone crest (CEJ-ABC) distances of maxilla with periodontitis mice (scale bar: 500 μm). Representative *X*-ray images (scale bar: 1.0 mm) **(C)** Gingipain (RgpA, KGP) expression analysis of gingival tissues from periodontitis mice by RT–PCR. GAPDH was used as the endogenous control in RT–PCR analysis of mouse specimens. Values are expressed as the mean ± SEM. The *p* value is indicated as follows: *p < 0.05, **p < 0.01, ***p < 0.001, ****p < 0.0001.

A periodontitis mouse model was constructed as previously described (Kimura et al., 2000; Huck et al., 2020). To induce periodontitis, the mouse was tied around the maxillary left second molar with a 6–0 silk ligature (Junsheng Co., CHN) and then orally infected with 0.2 mL of 5 × 10^10^ CFU/mL *P. gingivalis* four times every other day (Figure 1A). Fourteen days after the last infection, the mice were killed (using CO_2_), and the hemi-maxillae were collected and prepared for bone loss measurements. The heights of the hemi-maxillae were determined based on the distance between the cementoenamel junction (CEJ) and the alveolar bone crest (ABC) via microcomputer tomography (Micro-CT, see Supplementary material 2.3). The major *P. gingivalis* virulence factors RgpA and KGP were evaluated. The ligatures remained in place in all mice throughout the experimental period. Plasma was obtained by collecting blood and centrifuging it at 1000 × g for 10 min. Hemi-maxillae samples were collected in 10% neutral buffered formalin for histopathology. Gingival tissues were immediately processed into a single-cell solution for flow cytometry. Plasma and hemi-maxillae samples were stored at −20°C and room temperature, respectively. Eight mice were included in the test mouse model group, while untreated mice were used as controls (4 mice per group). The tools required for the procedure are depicted in the Supplementary material in Supplementary Table S1.

### Fibroblast cell culture

Human gingival fibroblasts (HGFs) were obtained from the BeNa Culture Collection (BNCC, China). HGFs were incubated in 90% Dulbecco’s Modified Eagle Medium (DMEM; Gibco, USA) supplemented with 10% fetal bovine serum (FBS; Sigma, USA) at 37°C in a humidified atmosphere containing 5% CO2 and 95% air.

### MTT assay: assessment of the optimal effect of *Porphyromonas gingivalis* on HGF infection

HGFs were seeded into 96-well plates at a density of 1 × 10^4^ cells per well and further incubated for 24 h. Then, the medium in the wells was replaced with *P. gingivalis* at an MOI of 100, 50, or 0. After incubation for 0 h, 4 h and 6 h, 10 μL of MTT solution (Cat# M1020, MTT Cell Proliferation and Cytotoxicity Assay Kit, Solarbio) was added into each well and the plates were further incubated for 4 h. After removing the medium containing MTT, 110 μL of DMSO (Cat# M1020, Solarbio) was added into each well to dissolve the formazan crystals with low-speed shaking for 10 min. The OD_490_ value was measured using a microplate reader. Untreated cells (MOI 0 for 0 h) were used as controls.

### Infection of HGFs with *Porphyromonas gingivalis*

HGFs were plated at 1 × 10^6^ cells/1 mL/well in triplicate into 6-well plates 1 d before infection, washed with phosphate-buffered saline (PBS; Solarbio, CHN), and exposed to *P. gingivalis* at an MOI of 100 for 6 h, 4 h, and 0 h. HGFs were infected with bacteria and then cultured at 37°C in a water-saturated atmosphere of 95% air and 5% CO_2_ for 0 h, 4 h, and 6 h. The culture supernatant was collected separately from triplicate wells for cytokine detection by enzyme-linked immunosorbent assay (ELISA), and total RNA was extracted from combined cells of triplicate wells using TRNzol Universal Reagent (TIANGEN, CHN). In addition, total protein was extracted for Western blotting analysis. For challenge with *P. gingivalis*, multiplicities of infection (MOIs; numbers of bacteria per mammalian cell) of 50 and 100 were used. Cells without any treatment (MOI 0 for 0 h) were used as controls.

### Preparation of gingival cell suspensions from gingival tissues

Gingival cells were isolated from mouse gingival tissues according to previous protocol with minor modifications (Mizraji et al., 2013; Jagannathan et al., 2014). Using collagenase D (1 mg/mL; Sigma–Aldrich) and DNase I (20 U/mL; Sigma–Aldrich), gingival tissues were minced and digested for 45 min at 37°C on a shaker. Next, tissues were passed through a 70-μm cell strainer, washed twice in complete RPMI 1640 medium (Gibco, Cat# 11875119), and centrifuged at 500 × g for 3 min at room temperature. Procedure details are provided in the Supplementary material 2.4. The cells were used for reactive oxygen species (ROS) assays and flow cytometry.

### Reactive oxygen species (ROS) assay

HGFs were seeded into 6-well plates at a density of 1 × 10^6^ cells per well and further incubated for 24 h. Then, prepared bacterial solutions at an MOI of 100 were added to the wells. The plates were incubated at 37°C, 5% CO2 and 95% humidity for 0 h, 4 h, and 6 h. 2′,7′-Dichlorodihydrofluorescein diacetate (DCFH-DA) (Cat# CA1410, 10 mM, Reactive oxygen species assay kit, Solarbio) was added to each well. Following 20 min of incubation at 37°C, fluorescence units were immediately measured with an excitation wavelength of 488 nm and an emission wavelength of 525 nm using a Thermo Varioskan LUX (Thermo, USA). Rosup (Cat# CA1410, Solarbio)-treated cells were used as a positive control. Gingival cell ROS were measured by means of the previously described cell DCFH-DA fluorescence technique, which can be used to track changes in the ROS concentration.

### ELISA for inflammatory cytokines

In accordance with the manufacturer’s instructions, ELISA kits were used to quantify the concentration of inflammatory cytokines in the supernatant of cultured cells (HGFs). Each sample was tested in duplicate in two or more replicate experiments. We calculated the values using recombinant cytokine standard curves. The results are given in picograms per milliliter (pg/mL) format. Supernatants of HGFs were analyzed for the presence of IL-6 (Cat# MM-0163 M1, MEIMIAN), IL-1β (Cat# MM-0040 M1, MEIMIAN), TNF-α (Cat# MM-0132 M1, MEIMIAN), and IFN-β (Cat# MM-0124 M1, MEIMIAN). To determine the inflammatory cytokine concentration, blood was collected from each mouse group 14 days after the operation. For further use, serum was centrifuged (15 min, 1,000 g) after coagulation (90 min at room temperature).

### Flow cytometry

The flow cytometry procedure was performed according to a previous protocol with minor modifications (Jiang et al., 2022a). Procedure details are also provided in the Supplementary material 2.5. We obtained single-cell suspensions from gingival tissues or spleen tissues. To examine macrophage polarization, cells were collected and adjusted to a density of 1 × 10^6^/mL with phosphate-buffered saline (PBS; Solabio, pH 7.4). Cells were first stained with Fixable Viability Stain 620 (BD Pharmingen, Cat# 564996) using the manufacturer’s recommended concentration of dye for 30 min at 4°C. Next, Fc receptor-specific antibody binding was blocked by adding rat anti-mouse CD 16/CD32 (BD Pharmingen, Clone: 2.4G, Cat# 553141) and incubating for 5 min at 4°C. The surface markers were stained with anti-CD206 (BD Pharmingen, clone: Y17-505, Cat# 568273), anti-F4/80 (Biolegend, clone: 16-10A1, Cat# 104729), anti-CD11c (Biolegend, clone: N418, Cat# 117338), and anti-CCR2 (BD Pharmingen, clone: BZ2E3, Cat# 743685) for 30 min at 4°C in a total volume of 100 μL. Then, we fixed the cells in 1% methanol-free formaldehyde (Solarbio, Cat# No. N1012) for 30 min at 4°C. After decanting the formaldehyde, we stained the intracellular antigens. We resuspended the cells in 1× permeabilization buffer (Invitrogen, Cat# 00–8,333-56) and incubated them for 5 min at RT. Then, the cells were stained with anti-CCL2 (R&D Systems, clone: 123616, Cat# IC479A) in a total volume of 100 μL for 30 min at 4°C. We used a flow cytometer (Beckman, USA) to evaluate all of the samples, and the results were processed in CytoFLEX software (Beckman, USA).

### Quantitative real-time PCR (RT–qPCR)

TRIzol Universal reagent (Cat#DP424, TIANGEN) was used to extract total RNA from cells and tissues. Total RNA quality and concentration were measured using a Thermo Scientific Varioskan Flash (Thermo Fisher Scientific, USA). From 1 μg of RNA, cDNA was synthesized. Real-time polymerase chain reaction (PCR) was performed with a QuantStudio 6 Flex (Life Technologies, USA) detection system and SYBR green I dye reagent (TaKaRa Biotechnology, Japan) in accordance with the manufacturer’s instructions. All specific primers (IFN-β, STING, cGAS, RANKL and GAPDH) were designed with Primer 5 software according to the sequences obtained from the GenBank database (Supplementary material S1). The relative gene expression for all genes was analyzed using the 2^-△△Ct^ method by normalization to GAPDH expression in all experiments.

### Western blotting

Total protein samples were collected from HGFs and tissues using RIPA buffer (Cat#R0010, Solabio) containing a protease inhibitor cocktail (Cat# No. P0100, Solabio). The BCA Protein Assay Kit (Cat# 23225, Thermo) was used to determine the protein concentrations. The proteins were separated by SDS–PAGE and transferred to polyvinylidene difluoride membranes (Millipore, Billerica, MA, USA). The antibodies used for this assay were anti-cGAS antibody (1:250, Cat# A8335, ABclonal), anti-STING rabbit mAb (1:500, Cat# A21051, ABclonal), anti-RANKL rabbit mAb (1:500, Cat# A2550, ABclonal), anti-β actin antibody (1:10000, Cat# 380624, Zen Biotechnology), and horseradish peroxidase-conjugated goat anti-rabbit IgG (1:1000, Cat# ab7090, Abcam). Pierce ECL Western blotting Substrate (Cat# P0018A-1, Beyotime) and a ChemiDoc Touch Imaging System (Bio-Rad, USA) were used to visualize and quantify the immunoblots.

### Histopathology

Procedure details (Supplementary material 2.6) for histopathology according to a previous protocol with minor modifications (Cardiff et al., 2014). In brief, maxillae from necropsied mice were preserved in 10% neutral buffered formalin, embedded in paraffin, and sectioned for *P gingivalis* detection and control at 3–5 mm for histopathologic examination. Then, hematoxylin and eosin (H&E) staining was performed following dehydration of the sections. The pathological sections were examined and photographed by a microscope (Leica, Germany). H&E-stained sections were used for histopathological analysis. An expert pathologist histologically scored each group. As described previously (Jiang et al., 2021), tissue damage severity was assessed using a semiquantitative scoring system. Several of histological indicators of injury were observed, including inflammatory cell infiltration, exudation, hemorrhage, and edema. In each category, scores ranged from 0 to 4 (0 = normal; 1 = injured area ≤ 25%; 2 = injured area 25–50%; 3 = injured area 51–75%; 4 = injured area > 75%). Semiquantitative analysis of H&E pictures was performed using Fiji software.

### Small molecule modulator treatment experiments

The *P. gingivalis*-induced periodontitis model was used as previously described in mice. Periodontitis mice were randomly distributed into different groups and intraperitoneally injected with SR-717 (30 mg/kg), SN-011 (10 mg/kg) or control (5% DMSO in PBS) for 7 days of daily dosing (*n* = 4). All modulators were dissolved in PBS with 5% DMSO (Cat# No. D2650, Sigma–Aldrich). After 7 days, the mice were sacrificed. Gingival tissues and serum were removed. Western blotting, H&E staining, ELISA and morphometric analysis were used to measure inflammatory cytokines and bone resorption changes. Untreated (5% DMSO in PBS) periodontitis mice served as controls.

### Statistical analysis

The results are shown as the mean ± SEM or GMTs, along with their 95% confidence intervals (CIs). An unpaired two-sided Student’s t test was utilized to determine statistical significance between two groups. One-way ANOVA with Tukey’s *post hoc* test was employed to assess differences among multiple groups. *p < 0.05, **p < 0.01, ***p < 0.001, ****p < 0.0001. The details on the statistical test and the error bars are specified in the figure legends. The results analysis and plotting were performed using GraphPad Prism 9.1.

## Results

### *Porphyromonas gingivalis* affects the viability of HGFs and induces the generation of ROS in HGFs

**Figure 2:**
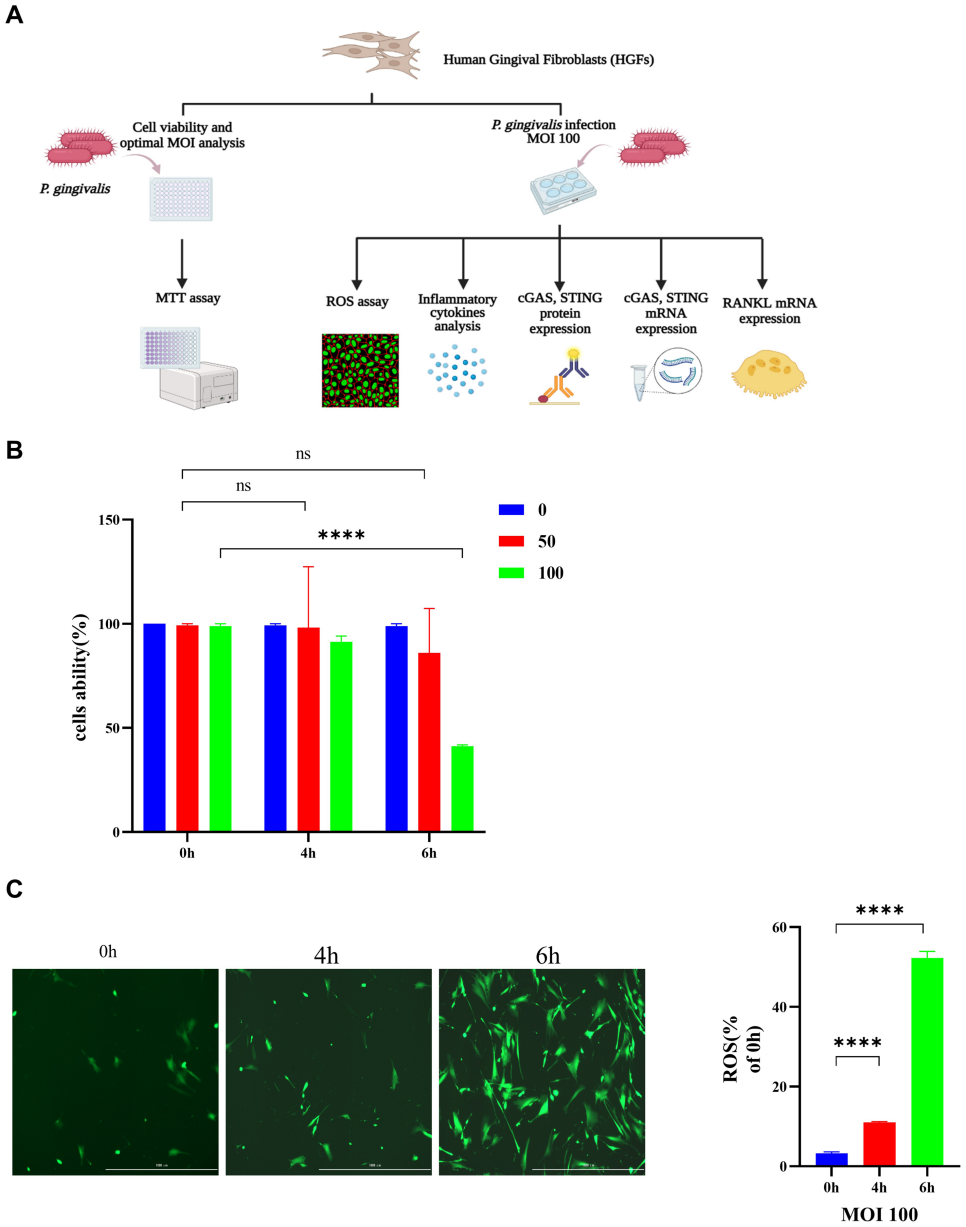
*Porphyromonas gingivalis* affects the viability of HGFs and induces the generation of ROS in HGFs. The cells were incubated with *P. gingivalis*. Cells without any treatment (MOI 0 for 0 h) were used as controls. **(A)** Diagram of the cell experimental procedure. **(B)** Cell viability after incubation with *P. gingivalis* at an MOI of 100, 50, or 0 for 0 h, 4 h, or 6 h as determined by MTT assay. **(C)** HGFs were preincubated with *P. gingivalis* (MOI 100 for 6 h) to detect ROS-induced fluorescence (scale bar, 1,000 μm). Statistical analysis of ROS-positive cells per field in the three groups. The data are expressed as the mean ± the SEM of four independent experiments (*n* = 4). The *p* value is indicated as follows: *p < 0.05, **p < 0.01, ***p < 0.001, ****p < 0.0001. ns, no significance; MOI, multiplicity of infection; ROS, reactive oxygen species.

The role of *P. gingivalis* in HGFs was explored initially. A diagram of the experimental procedure is depicted in Figure 2A. As shown in Figure 2B, cell survival was significantly decreased by *P. gingivalis* treatment at an MOI of 100 for 6 h compared with the control cells (49.7%) (*p* < 0.0001), but HGF cell viability was not affected at an MOI of 50 for 0 h and 4 h. HGFs infected with *P. gingivalis* may have increased production of inflammatory cytokines and increased expression of iNOS (inducible nitric oxide synthase), which may have also increased the accumulation of NO and ROS, resulting in an increased proportion of HGFs undergoing apoptosis. Thus, *P. gingivalis* at an MOI of 100 was used in subsequent experiments for further mechanistic investigations. Additionally, incubation with *P. gingivalis* (MOI 100) for 4 h or 6 h resulted in intracellular ROS levels that were significantly elevated compared to those in uninfected cells (0 h), as demonstrated by fluorescence microscopy (*p* < 0.0001, Figure 2C).

### Exposure to *Porphyromonas gingivalis* activates the cGAS-STING signaling pathway *in vitro*

**Figure 3:**
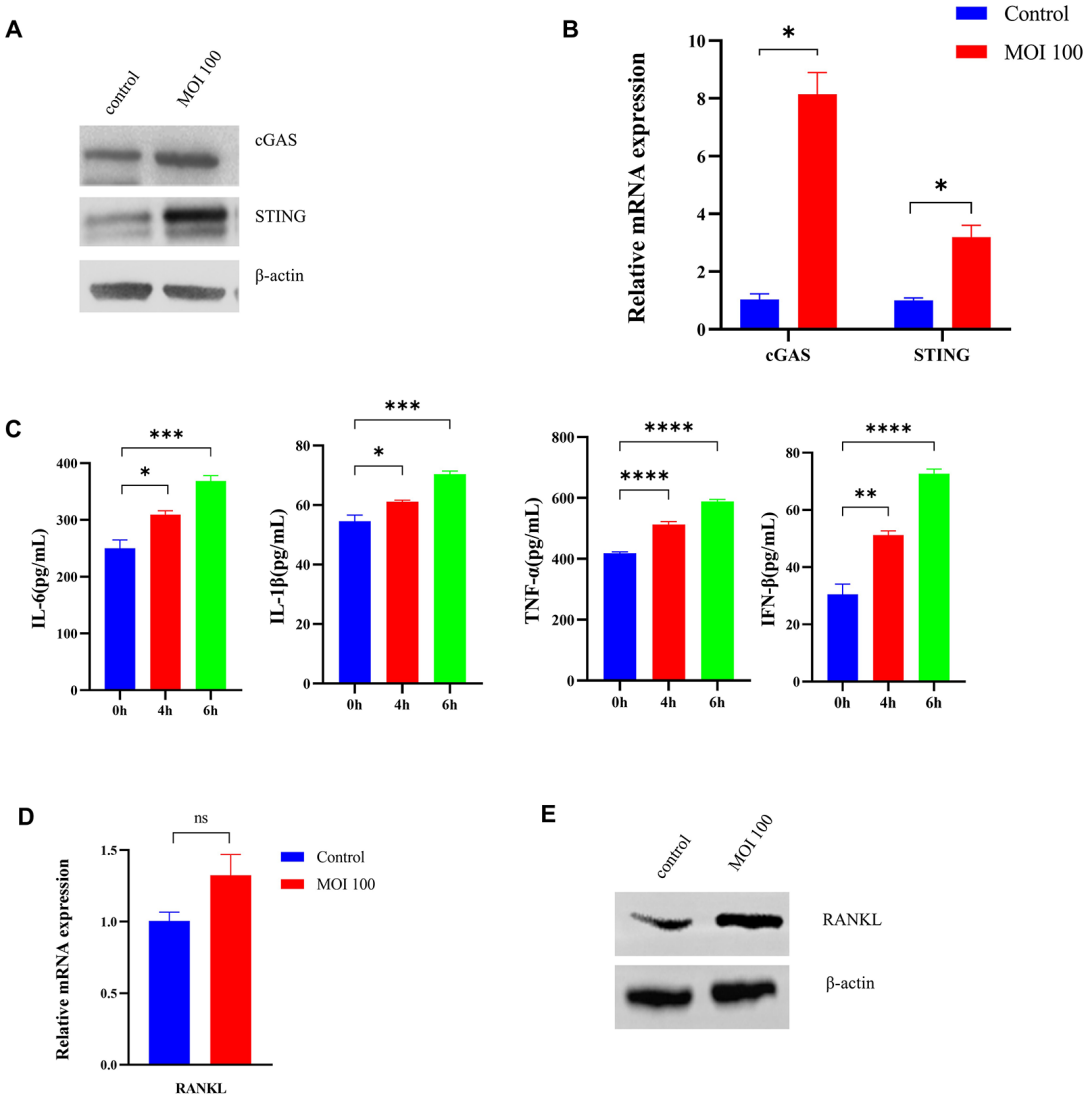
*Porphyromonas gingivalis* activates the cGAS-STING signaling pathway *in vitro*. HGFs were infected with *P. gingivalis* at an MOI of 100 for 6 h. Untreated cells (MOI 0 for 0 h) were used as controls. **(A)** Total lysates were harvested, and cGAS and STING protein levels in HGFs were confirmed by Western blotting. β-Actin served as a control. **(B)** Transcript levels of cGAS and STING were measured by qRT–PCR and normalized to uninfected cells under each treatment using the threshold cycle (2^−ΔΔCt^) method with GAPDH as the reference gene. **(C)** Supernatants were collected, and the levels of IL-1β, IL-6, TNF-a, and IFN-β were analyzed by ELISA. **(D)** Cells were also collected to measure the gene expression of RANKL by qRT–PCR. **(E)** Western blotting analysis of RANKL expression in HGFs. Values are expressed as the mean ± SEM (*n* = 4). The *p* value is indicated as follows: *p < 0.05, **p < 0.01, ***p < 0.001, ****p < 0.0001. ns, no significance.

To explore the role of activation of the cGAS-STING pathway in periodontopathogenic bacteria-induced experimental periodontitis, we examined the effect of *P. gingivalis* on infection *in vitro* (Figure 3A). The expression of cGAS and the STING protein in HGFs stimulated by *P. gingivalis* at an MOI of 100 for 6 h was analyzed by Western blotting analysis (Figure 3A) and RT–qPCR (Figure 3B). The cGAS and STING levels in HGFs were detected after treatment with *P. gingivalis* for 6 h (Figure 3A). Additionally, the mRNA levels of cGAS and STING in the cultured cells increased dramatically at 6 h and were 8-and 3-fold higher than the levels in control HGFs, respectively (*p* < 0.05, Figure 3B).

Moreover, we measured the levels of inflammatory cytokines (IL-6, IL-1β, TNF-a, IFN-β) secreted from HGFs treated with *P. gingivalis* by ELISA. As shown in Figure 3C, *P. gingivalis* significantly promoted inflammatory cytokine production in a time-dependent manner in HGFs (*p* < 0.05). To explore RANKL expression under *P. gingivalis* conditions, the level of RANKL in HGFs was examined. As shown in Figure 3D, RANKL mRNA levels were higher than that of the control group, but the difference was not significant. The same phenomenon was also observed at the protein level by western blotting (Figure 3E). These data are in line with changes in proinflammatory cytokines, generally considered downstream effectors in the STING-mediated immune response. The above studies suggested that *P. gingivalis* activates the cGAS-STING pathway and induces inflammatory cytokine expression under *in vitro* conditions.

### The *Porphyromonas gingivalis* infection-driven cGAS-STING signaling pathway induces type I IFN gene expression in mouse models of periodontitis

To gain insight into the events underlying the *P. gingivalis* mechanisms of periodontitis, we constructed a periodontitis mouse model (WT C57BL/6) and evaluated the ROS levels of gingival cells, gingipain (RgpA, KGP) expression, CEJ-ABC distance, cGAS and STING expression, proinflammatory cytokine secretion, and RANKL mRNA expression (Figure 1A). First, we observed that the periodontitis model animals had significantly (*p* < 0.05) more CEJ-ABC distances and had a greater reduction in alveolar bone volume in contrast to the control group; furthermore, model animals had a higher level of gingipain (RgpA, KGP) expression (*p* < 0.001), as determined by micro-CT (Figure 1B) and RT–PCR (Figure 1C).

**Figure 4:**
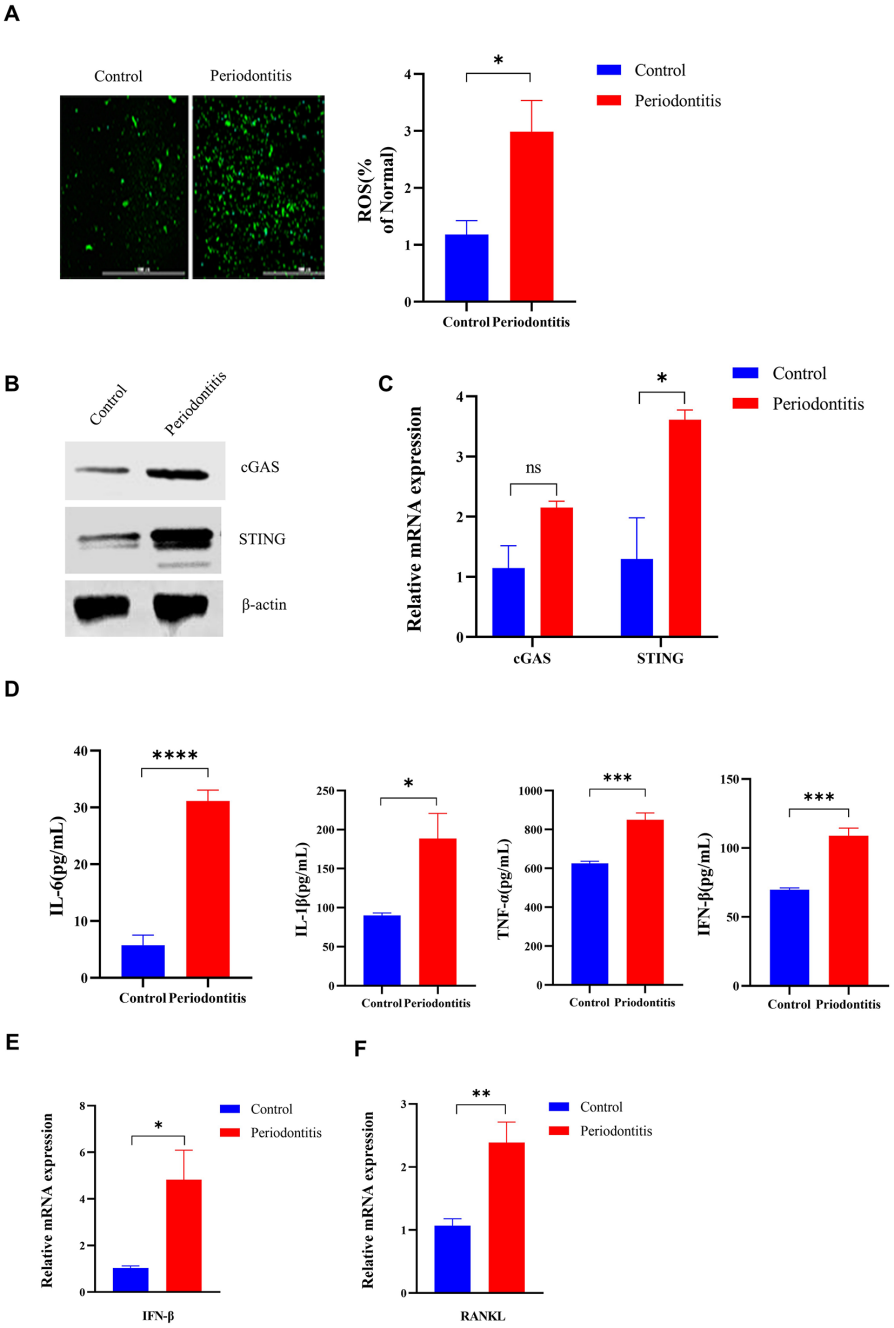
The *Porphyromonas gingivalis* infection-driven cGAS-STING signaling pathway induces type I IFN gene expression in mouse models of periodontitis. The periodontitis model was established using C57BL/6 mice, and untreated animals served as the control group (*n* = 4 mice per group). **(A)** Mouse gingival cells were preincubated with DCFH-DA (10 mM) to detect ROS-induced fluorescence. Statistical analysis of ROS-positive cells per field in the two groups. Uninfected mice were used as controls (scale bar, 1,000 μm). **(B)** Mouse gingival tissue lysates were collected to determine the levels of cGAS and STING by Western blotting. **(C)** Mouse gingival tissue was collected to measure the gene expression of cGAS and STING by qRT–PCR. **(D)** Serum samples were collected, and the levels of IL-6, IL-1β, TNF-a and IFN-β were analyzed by ELISA. **(E,F)** Mouse gingival tissues were also collected to measure the gene expression of IFN-β **(E)** and RANKL **(F)** by qRT–PCR. Values are expressed as the mean ± SEM. The *p* value is indicated as follows: *p < 0.05, **p < 0.01, ***p < 0.001, ****p < 0.0001. ns, no significance.

In the periodontitis mouse model, the ROS levels in the gingival cells of periodontitis mice were markedly increased compared with those in the control mice (*p* < 0.05, Figure 4A). In addition, cGAS and STING protein expression was detected by Western blotting (Figure 4B). Moreover, the mRNA levels of cGAS and STING in periodontitis mice were 2-and 3-fold higher than those in the control group, respectively (Figure 4C). Compared with the control group, periodontitis mice exhibited a significant increase in proinflammatory cytokine (IL-6, IL-1β, TNF-a, IFN-β) production (*p* < 0.05, Figure 4D). Moreover, the periodontitis mouse model group had IFN-β expression levels ∼5-fold higher than those in the control group (*p* < 0.05, Figure 4E). Then, we observed an increased expression of RANKL in gingival tissues compared to that of the control group by RT–qPCR, with a 2-fold increase (*p* < 0.01, Figure 4F). These results indicated that *P. gingivalis* infection activates the cGAS-STING pathway, resulting in the production of type I IFN and subsequent production of different inflammatory cytokines in a mouse model of periodontitis, all of which contribute to the formation and activation of osteoclasts.

### *Porphyromonas gingivalis* induces significantly lower type I IFN gene expression in periodontitis Sting^Gt^ mice

**Figure 5:**
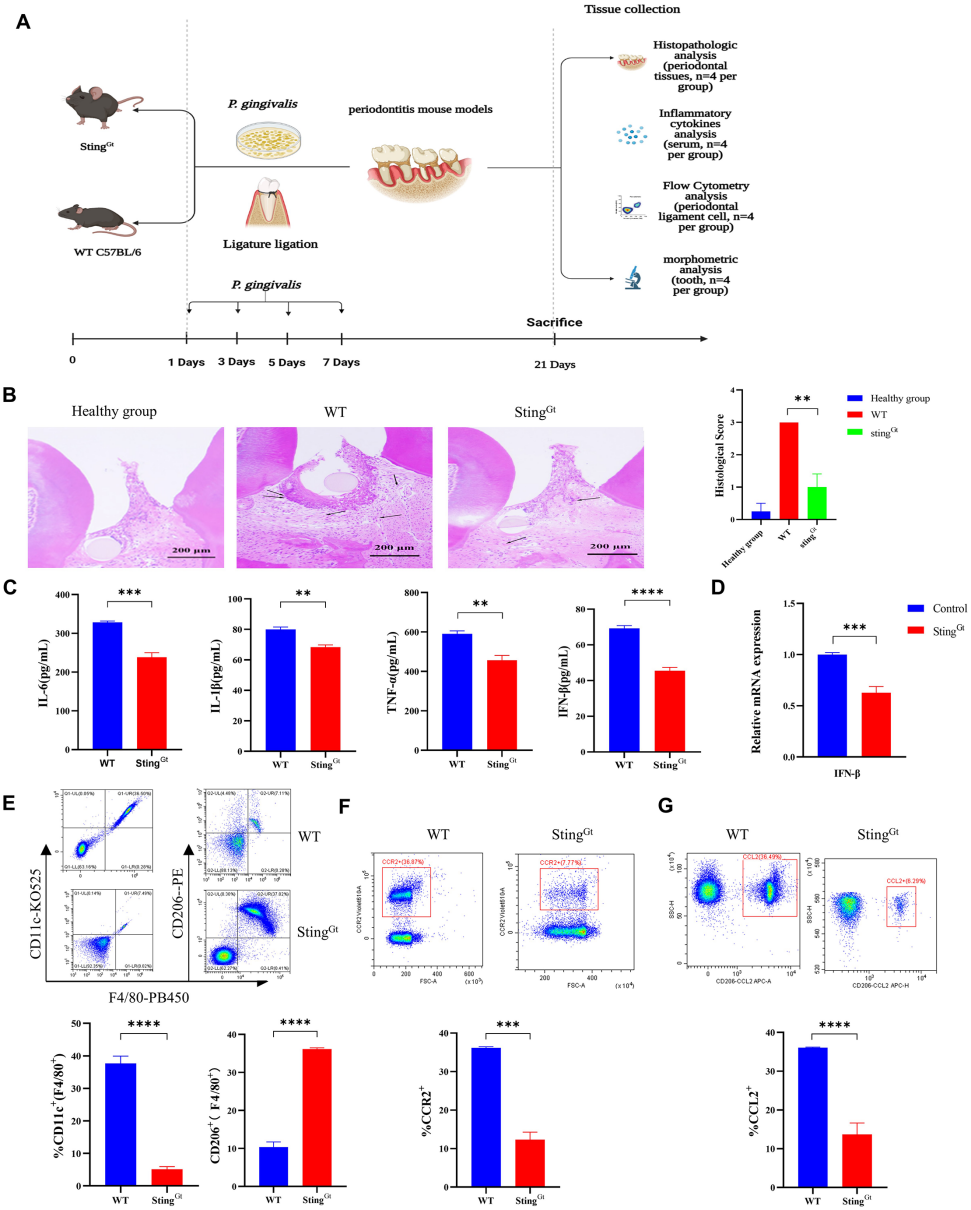
*Porphyromonas gingivalis* decreases proinflammatory cytokine synthesis in periodontitis Sting^Gt^ mice via the cGAS-STING signaling pathway. Sting^Gt^ and C57BL/6 mice were used to construct periodontitis models, with C57BL/6 periodontitis mice serving as controls (*n* = 4 mice per group). **(A)** Diagram of the STING transgenic (Sting^Gt^) mouse periodontitis model. **(B)** Images showing H&E staining following infection with *P. gingivalis*. The images shown are 14 dpi for all groups (scale bar, 200 μm). Each image is representative of a group of 4 mice at 14 dpi. Semiquantitative analysis of H&E pictures was also performed. **(C)** Serum samples were collected, and the levels of IL-6, IL-1β, TNF-a and IFN-β were analyzed by ELISA. **(D)** Mouse gingival tissues were also collected to measure the gene expression of IFN-β by qRT–PCR. **(E–G)** The gingival cells were harvested and stained with mAbs specific for F4/80, CD11c, CD206, CCR2, and CCL2 for flow cytometric analysis. The results are expressed as M1 macrophages: F4/80 + CD11c+; M2 macrophages: F4/80 + CD206+. The relative proportion of M1 macrophages and M2 macrophages is shown in **(E)**. The relative proportion of CCR2 **(F)** and CCL2 **(G)** in gingival cells induced by *P. gingivalis*. The results shown are 14 dpi for all groups. Data are expressed as the mean ± SEM. The *p* value is indicated as follows: *p < 0.05, **p < 0.01, ***p < 0.001, ****p < 0.0001. ns, no significance.

To evaluate whether the STING gene could affect *P. gingivalis* infection-induced type I IFN gene expression, we constructed a periodontitis model in Sting^Gt^ mice and then compared the pathological changes, proinflammatory cytokine activity, macrophage polarization and bone resorption between Sting^Gt^ mice and WT C57BL/6 periodontitis mice (4 mice per group; Figure 5A). First, we evaluated the pathological changes in periodontitis mice and observed large amounts of inflammatory cell infiltration in all WT periodontitis groups, with a slight response in Sting^Gt^ mice (*p* < 0.01, Figure 5B), more H&E images are shown in Supplementary Figure S1. Then, we addressed whether proinflammatory cytokine activity could be altered by STING knockout. As shown in Figure 5C, the levels of IL-6, IL-1β, TNF-α, and IFN-β secreted under the influence of *P. gingivalis* were lower in the Sting^Gt^ periodontitis mice than in the WT periodontitis mice (*p* < 0.05). Moreover, periodontitis Sting^Gt^ mice had IFN-β expression levels ∼1.6-fold lower than those in WT mice (*p* < 0.001, Figure 5D).

**Figure 6:**
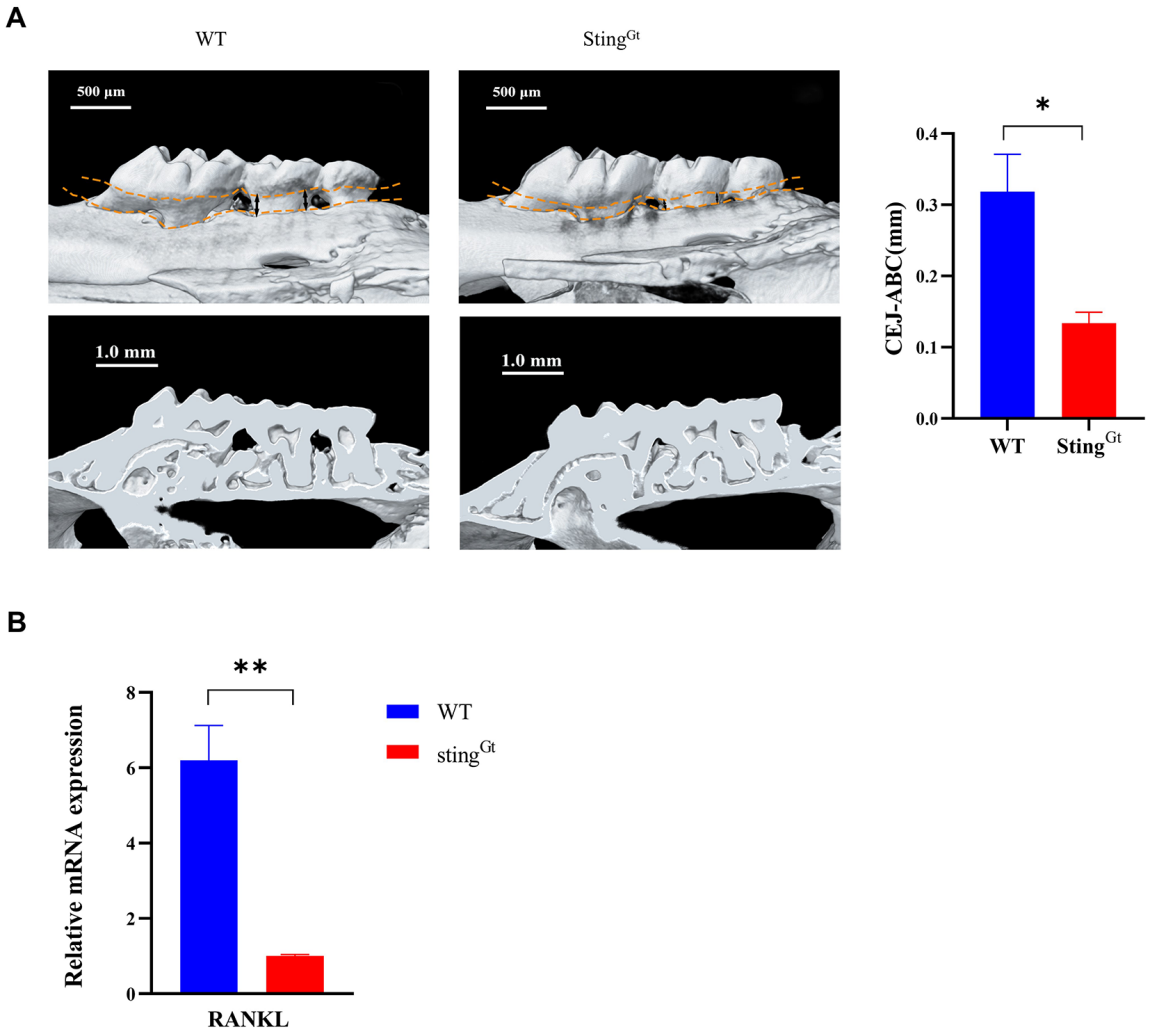
*Porphyromonas gingivalis* decreases alveolar bone loss in periodontitis Sting^Gt^ mice via the cGAS-STING signaling pathway. **(A)** Three-dimensional reconstruction images and analysis of the cementoenamel junction to alveolar bone crest (CEJ-ABC) distances of maxilla with periodontitis mice (scale bar: 500 μm). Representative X-ray images (scale bar: 1.0 mm) **(B)** Mouse periodontal tissues were also collected to measure the gene expression of RANKL by qRT–PCR.

Furthermore, we used Sting^Gt^ mice to explore the role of the cGAS-STING pathway in macrophage polarization under periodontitis conditions. Flow cytometry showed that the Sting^Gt^ group manifested significantly enhanced F4/80+ macrophage infiltration, increased CD206+ (M2 marker) cell populations, and decreased CD11c + (M1 marker) cell populations compared with the WT group (*p* < 0.0001, Figure 5E). In addition, flow cytometry also showed significantly decreased chemokine (CCR2 and CCL2) levels in gingival tissues from periodontitis mice in the Sting^Gt^ group in comparison with that in the WT group (*p* < 0.001, Figures 5F,G). Additionally, we observed that Sting^Gt^ periodontitis mice had reduced CEJ-ABC distances (*p* < 0.05), had less alveolar bone volume loss than WT mice, and had a lower level of RANKL expression (*p* < 0.01) based on micro-CT (Figure 6A) and RT–PCR (Figure 6B). All these data suggested that the level of type I IFN decreased with knockout of the STING gene via the cGAS-STING pathway during *P. gingivalis* infection.

### Suppression of cGAS/STING ameliorates *Porphyromonas gingivalis*-induced experimental periodontitis

**Figure 7:**
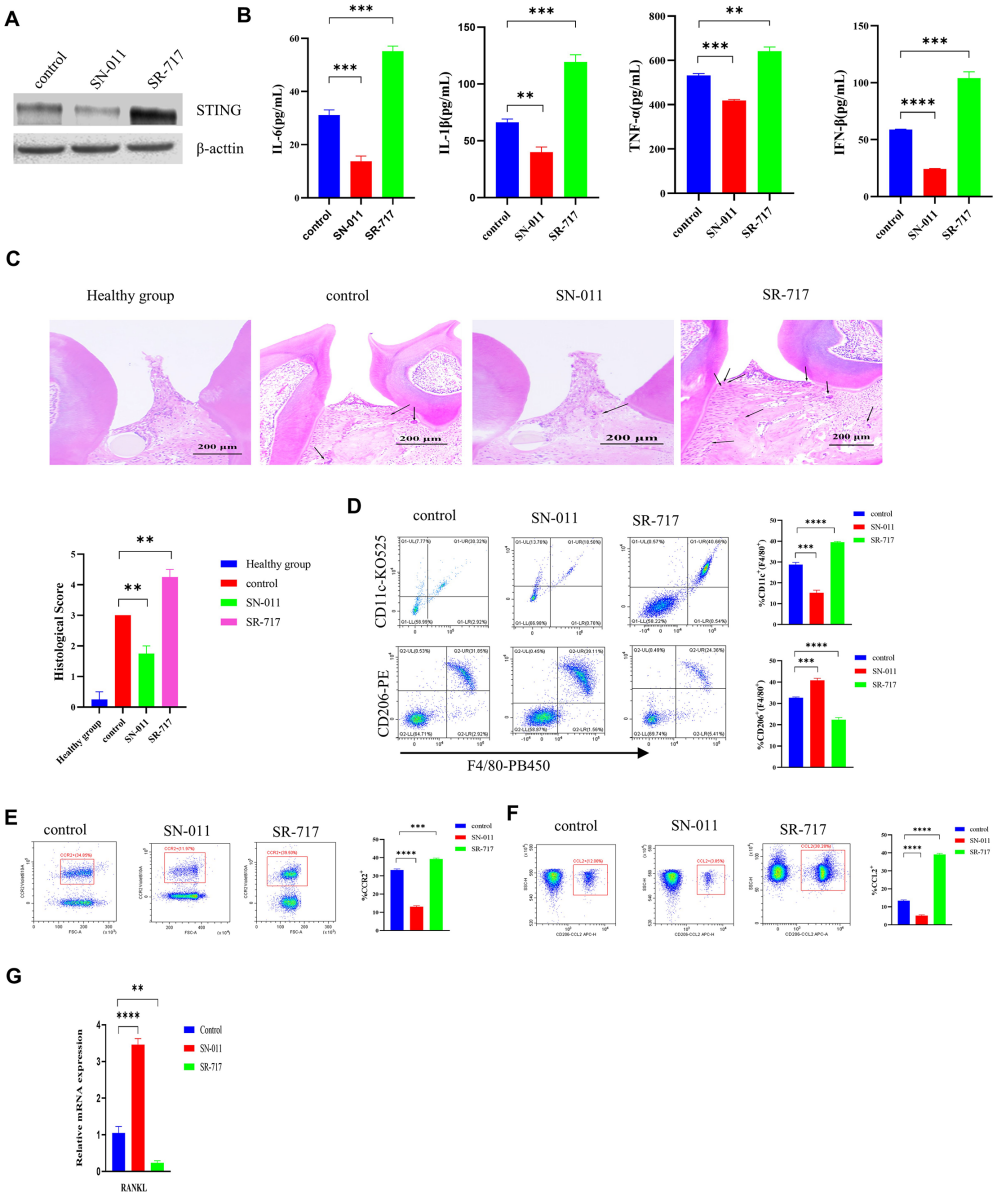
Suppression of cGAS/STING ameliorates *Porphyromonas gingivalis*-induced periodontal disease. *P. gingivalis*-induced periodontitis mouse groups were injected intraperitoneally with SR-717 (30 mg/kg), SN-011 (10 mg/kg) and vehicle (5% DMSO in PBS) for 7 days of daily dosing and euthanized after the last treatment. Mice with PBS-treated periodontitis served as controls (*n* = 4 mice per group). **(A)** Mouse gingival tissue lysates were collected to determine the protein levels of cGAS and STING by Western blotting. **(B)** Serum samples were collected, and the levels of IL-6, IL-1β, TNF-a and IFN-β were analyzed by ELISA. **(C)** H&E staining showed hemi-maxillae histopathological changes after treatment with small molecule modulators (scale bar, 200 μm). Semiquantitative analysis of H&E pictures was also performed. **(D–F)** Gingival cells were harvested and stained with mAbs specific for F4/80, CD11c, CD206, CCR2, and CCL2 for flow cytometric analysis. The results are expressed as M1 macrophages: F4/80 + CD11c+; M2 macrophages: F4/80 + CD206+. The relative proportion of M1 macrophages and M2 macrophages is shown in **(D)**. The relative proportion of CCR2 **(E)** and CCL2 **(F)** in periodontal ligament cells induced by *P. gingivalis*. **(G)** Mouse gingival tissues were also collected to measure the gene expression of RANKL by qRT–PCR. Data are expressed as the mean ± SEM. The *p* value is indicated as follows: *p < 0.05, **p < 0.01, ***p < 0.001, ****p < 0.0001. ns, no significance.

The STING inhibitor SN-011 or agonist SR-717 was employed to evaluate the effect of STING on regulating the *P. gingivalis*-induced activation of the cGAS-STING pathway in WT C57BL/6 periodontitis mouse model. Our data showed that the SR-717 group had greater levels of STING than the control group, as evaluated by Western blotting; however, the SN-011 group showed lower expression of STING than the control group (Figure 7A). Then, higher levels of inflammatory cytokines were detected in the SR-717-treated group, which corresponded with lower inflammatory cytokine levels in the SN-011-treated group compared to the control group, as determined by ELISA (*p* < 0.01, Figure 7B). Additionally, H&E staining showed a significant decrease in the number of inflammatory cells in the SN-011-treated group compared to the control group. However, inflammatory cell infiltration was more abundant in SR-717-treated mice than in the control group mice (*p* < 0.01, Figure 7C), more H&E images are shown in Supplementary Figure S2.

Flow cytometry showed that the SR-717-treated group had a significantly elevated number of M1 macrophages (CD11c + F4/80+) and a decreased number of M2 macrophages (CD206 + F4/80+) compared with the control group (*p* < 0.001, Figure 7D). Compared with the control group, the SN-011-treated group had reduced M1 macrophage polarization and increased M2 macrophage polarization (Figure 7D). In comparison with the control group, the SR-717-treated group showed significantly upregulated levels of chemokines (CCR2 and CCL2), whereas the level of chemokines decreased in the SN-011-treated group (*p* < 0.001, Figures 7E,F). We also observed that SR-717-treated mice had a higher RANKL expression, whereas SN-011-treated mice exhibited lower RANKL expression compared to control mice (*p* < 0.01, Figure 7G). These results indicated that SN-011 significantly inhibited *P. gingivalis*-induced periodontitis.

## Discussion

*Porphyromonas gingivalis* is a major pathogen associated with chronic periodontitis that is estimated to affect >10% of the adult population worldwide (Eke et al., 2020). Identification of small molecule modulators that reduce excessive inflammatory responses to *P. gingivalis* will facilitate the development of novel therapies and may improve responses to currently available therapies. In 2022, Uemura et al. (2022) showed that *Pg*-OMV induces inflammatory cytokines to be released from gingival epithelial cells and suggested that *Pg*-OMVs may play important roles in periodontitis exacerbation by stimulating various pathways *in vitro*. However, it remains unknown whether and how *P. gingivalis* is involved in regulating the cGAS-STING signaling pathways in gingival tissues and its role in periodontitis pathogenesis. In the present study, we first found that *P. gingivalis* infection triggers type I IFN gene and related cytokine expression and leads to activation of the cGAS-STING pathway both *in vitro* and in a periodontitis mouse model. Then, we verified that Sting^Gt^ mice showed lower levels of inflammatory cytokines and bone resorption than wild-type periodontitis-infected mice. Finally, we observed that STING inhibitors and agonists can change periodontitis progression. These results suggest that *P. gingivalis* infection may activate the immune response and inflammation through the cGAS-STING pathway in periodontitis.

In a study by Elmanfi et al. (2021), immunohistochemical analyses indicated that in periodontitis, strong STING accumulation was detected in the basal epithelium and around vessel walls in the connective tissue; however, STING was weakly present in healthy gingiva, similar to what we observed in our *P. gingivalis in vitro* HGF infection model. In the present study, we found that *P. gingivalis* increases the expression of cGAS and STING and increased proinflammatory cytokine release *in vitro*, which is in line with earlier findings. It has been reported that gingival fibroblasts express cGAS and STING and produce various inflammatory cytokines, including IL-6, IL-8, and CCL2, in periodontitis (Mankan et al., 2014; Kitaura et al., 2020). These data suggested that *P. gingivalis* infection leads to an increase in cGAS and STING expression, as well as an increase in inflammatory cytokines *in vitro*. Thus, to further address the role of the cGAS-STING pathway *in vivo*, we established mouse models of *P. gingivalis* infection periodontitis and observed that periodontitis mice exhibited higher levels of cGAS and STING protein and secreted more inflammatory cytokines than control mice. Next, we proved that STING is crucial for *P. gingivalis* infection since STING KO mice exhibited lower levels of inflammatory cytokines than control (WT) mice. Along these lines, the absence of STING reduced the inflammatory response and reduced alveolar bone loss. Treatment of periodontitis mice with SR-717 resulted in significantly higher expression of inflammatory cytokines compared to the control group, while treatment of mice with SN-011 resulted in significantly lower levels of inflammatory cytokines. Hence, SN-011 is regarded as a competitive antagonist that inhibits the cGAS-STING signaling pathway and suppresses the innate inflammatory response. Our current findings support the notion that the cGAS-STING pathway activates the transcription and expression of genes encoding type I IFNs (e.g., IFN-β) and various cytokines and chemokines (e.g., IL-6 and TNF; Herath et al., 2013). STING-activated type I IFNs are key cytokines that are induced by antimicrobial and antiviral immunity (Ablasser and Gulen, 2016). Mirroring this finding, we found that *P. gingivalis* infection also leads to the production of type I IFNs and various cytokines through the cGAS–STING pathway.

Various inflammatory cytokines play roles in the development and activation of osteoclasts. Ay et al. (2012) demonstrated that IL-1, IL-6, IL-11, IL-17, and TNF-α, generated by infiltrating immune cells, induce bone resorption in periodontitis. These inflammatory cytokines stimulate osteoclastogenesis (Kitaura et al., 2020). In the current study, we found that mice with periodontitis showed significant alveolar bone resorption compared to control mice after *P. gingivalis* infection. On the other hand, Sting^Gt^ periodontitis models mice exhibited reduced alveolar bone loss. The reason for this may be that the deficiency of STING in *P. gingivalis*-infected mice prevented the inflammatory response, which in turn could be responsible for the lower level of proinflammatory cytokines needed to activate osteoclast differentiation and evoke less bone loss; however, further investigation is needed. In addition, bone loss was prevented in RANKL-deficient mice with periodontitis, suggesting that osteoclastogenesis in periodontitis is dependent on RANKL (Feng et al., 2019). Takahashi et al. (2014) found an increase in RANKL expression after the induction of experimental periodontitis by ligation. Gingival fibroblasts also express RANKL after challenge with periodontopathogens such as *P. gingivalis* (Wielento et al., 2023). In the present study, we assessed RANKL expression in gingival tissue samples from experimental periodontitis mice. Similar to Cantley et al. (2011), we observed a positive correlation between bone loss and the expression of RANKL in gingival tissue. The present results agree with those of Deng et al. (2020), who showed that RANKL determines the rate of alveolar bone loss and the progression of periodontitis.

Periodontitis causes alveolar bone destruction in the gingival tissue along with extensive macrophage infiltration (Liu et al., 2023). Macrophages are both heterogeneous and plastic, have polarized phenotypes and exhibit different functions depending on their environment (Zhu et al., 2019). Prior studies have noted the role of an unbalanced M1/M2 ratio in periodontitis-associated bone resorption (Zhang et al., 2021). Macrophage phenotypes could be altered during the progression of periodontitis, with increased M1 activation during the inflammation phase and repolarization toward M2 macrophages during the healing stage (Sun et al., 2020). Here, we reported that STING knockout inhibited M1 polarization and promoted M2 polarization, and intraperitoneal injection with the STING inhibitor SN-011 diminished the M1/M2 ratio and mitigated alveolar bone resorption in a mouse periodontitis model. Thus, we inferred that SN-011 promotes macrophage polarization toward the M2 phenotype and decreases bone resorption during the inflammatory process. However, Yamaguchi et al. (2016) found that M1 macrophages limit osteoclastogenesis by secreting IFN and IL-12, while M0 and M2 macrophages have little effect on osteoclast development. This phenomenon may be attributed to differences in experimental methods, such as the time of osteoclast induction. In our study, we discovered that compared to control mice, periodontitis mice exhibited macrophage polarization toward the M1 phenotype. Given the healing capacity of M2 macrophages, these findings imply that SN-011 may have a favorable influence on inflammation resolution in periodontitis. Further studies are required to investigate the effects of SN-011 on the alveolar bone regeneration and periodontal tissue repair during the treatment of periodontitis.

It was reported that macrophages play an important role in the pathogenesis of periodontitis, while CCL2-CCR2 is the major signal responsible for the recruitment of macrophages (Lira-Junior et al., 2020). In a study by Jiang et al. (2022b), the results showed that CCL2 and CCR2 may play an important role in the development of periodontitis. In our study, we observed that the levels of CCL2 and CCR2 were significantly decreased in Sting^Gt^ periodontitis mice compared with WT periodontitis mice. As expected, in contrast to untreated periodontitis mice, SR-717-treated mice had elevated CCL2 and CCR2 levels, while SN-011-treated mice had decreased levels. Our findings suggest that CCL2 and CCR2 may be involved in periodontitis progression by promoting macrophage recruitment, proinflammatory cytokine production and osteoclast formation.

However, our study has several limitations. First, research on the pathogenesis of periodontitis has only concentrated on the primary pathogen, *P. gingivalis*, and the effects of the existence and constituents of other indigenous bacteria have not been extensively explored. Second, we believe that it will be more meaningful to use cGAS-and IFNAR1-deficient mice together to study the impact of *P. gingivalis* on the pathogenesis of periodontitis. In future investigations, we will continue to test our hypotheses using cGAS-and IFNAR1-deficient mice. Third, although our work offered insight into cGAS/STING-mediated innate immune responses under periodontitis, the connections between individual pathways remain unknown. Fourth, while our method demonstrated that *P. gingivalis* stimulated HGFs to produce inflammatory cytokines, there was also partial apoptosis, and we need to evaluate the effect of apoptosis on inflammatory cytokines. Finally, research on the antibacterial role of the cGAS-STING pathway and its interaction with inflammation is worth further exploration.

In summary, we have demonstrated that *P. gingivalis* infection contributes to the progression of the inflammatory response via the cGAS-STING signaling pathway in HGFs and a mouse model of periodontitis. Furthermore, these findings indicated that the absence of STING may have prevented an inflammatory response, which could be responsible for a lower level of cytokines that inhibit osteoclast formation and induce less bone loss.

## Data availability statement

The original contributions presented in the study are included in the article/Supplementary material, further inquiries can be directed to the corresponding authors.

## Ethics statement

The animal study was reviewed and approved by the Committee on Ethics of the Institute of Medical Biology, Chinese Academy of Medical Sciences (IMBCAMS; assurance number: DWSP201912001).

## Author contributions

LS and JLL: conceptualization and methodology. RB, YY, HL, YM, LC, JYL, and GJ: investigation. RB and MS: data curation. RB: writing—original draft. MS and LS: writing—review and editing. LS and JY: funding acquisition. All authors contributed to manuscript revision and read and approved the submitted version.

## Funding

This study was funded by the Yunnan Provincial Science and Technology Department (Grant number 202002AA10009) and the Special Funds for High-Level Health Talents of Yunnan Province (Grant number L-201615). The funding source had no involvement in the study design; in the collection, analysis and interpretation of data; in the writing of the report; or in the decision to submit the article for publication.

## Conflict of interest

The authors declare that the research was conducted in the absence of any commercial or financial relationships that could be construed as a potential conflict of interest.

## Publisher’s note

All claims expressed in this article are solely those of the authors and do not necessarily represent those of their affiliated organizations, or those of the publisher, the editors and the reviewers. Any product that may be evaluated in this article, or claim that may be made by its manufacturer, is not guaranteed or endorsed by the publisher.
