# Supplementary material for: Porphyromonas gingivalis induces an inflammatory response via the cGAS-STING signaling pathway in a periodontitis mouse model
Source: Front Microbiol. 2023 Jun 19;14:1183415. doi: 10.3389/fmicb.2023.1183415 (PMC10315844; doi:10.3389/fmicb.2023.1183415)
Supplement: Supplementary file 1 [file Data_Sheet_1.PDF]

Supplementary Material

1 Sequences of primers for RT-qPCR

Table 1 Sequences of primers for RT-qPCR

Gene	Primers
GAPDH	Forward:5'TGTGTCCGTCGTGGATCTGA'3
	Reverse:5'CCTGCTTCACCACCTTCTTGA'3
cGAS	Forward:5'AGAAGGACTACCTATTCAAGGCT'3
	Reverse:5'GGGTACGAGATAAAACGGCTC'3
STING	Forward:5'TCGCACGAACTTGGACTACTG'3
	Reverse:5'CCAACTGAGGTATATGTCAGCAG'3
RANKL	Forward:5'CAGCATCGCTCTGTTCCTGTA'3
	Reverse:5'CTGCGTTTTCATGGAGTCTCA'3
RgpA	Forward:5'GTTCCATCACCGCTACCCAT'3
	Reverse:5'GGACAAGGACCGACGAAAGA'3
KGP	Forward:5'GACCCTGCGTTGTAGCAGT'3
	Reverse:5'GGTGTTGCTAATGCCAGCG'3
IFN-β	Forward:5'CTTCTCCACCACAGCCCTCTC'3
	Reverse:5'CCCACGTCAATCTTTCCTCTT'3

2 MATERIALS AND METHODS

2.1 Sting^{Gt} mice

Sting^{Gt} (Goldenticket, Tmem173gt) is a chemically-induced (ENU) mutant allele of the transmembrane protein 173 locus (Tmem173 or Sting) harboring a missense mutation in exon 6 - which results in an isoleucine-to-asparagine change in amino acid 199 in the C-terminal of the protein.

2.2 Ligature ligation and *P. gingivalis*–induced periodontitis mouse model

The tools required for the procedure are depicted in Supplementary Table S1.

2.3 Micro-CT

The sagittal plane of the specimens was set parallel to the x-ray beam axis. The specimens were scanned at a resolution of 12 µm in all three spatial dimensions. The scans were Gaussian filtered and segmented using a multilevel global thresholding procedure for the segmentation of enamel, dentin, and bone. Residual alveolar bone volume was determined separately for either root (bucco-mesial and bucco-distal) using a direct 3-dimensional approach.19 The measured mesio-distal length of the alveolar bone was 204 µm and 120 µm for the mesio-buccal and the disto-buccal roots, respectively. The apical basis of the measured volume was set mesio-distally parallel to the cemento-enamel junction (CEJ) and bucco-palatinally parallel to the occlusal plane. The results represented the residual bone above the reference plane in mm3. After micro-CT analysis was carried out, the hemi-maxilla was cleaned with hot water, 3% hydrogen peroxide, and 0.1% hypochlorite and stained with 1% methylene blue. Seven linear (millimeter) measurements were obtained from the maxillary molars of each jaw by using a stereomicroscope with an onscreen computer-aided measurements package. The results represented the distance between the CEJ and the alveolar bone crest (ABC) in millimeters (Wilensky et al. 2005). All alveolar bone loss measurements were performed by blinded examiners.

2.4 Preparation of gingival cells suspensions from gingival tissues

Gingival cells were isolated from mouse gingival tissues as previously described (McCulloch, Knowles, and Overall 1987; Jagannathan, Lavu, and Rao 2014). Tissue samples were immersed in RPMI 1640 at room temperature within 1 min following excision. Subsequent processing was completed within 30 min. The tissues were weighed, minced into very small pieces (< 1 mm^3), washed 3 times in RPMI 1640 and then placed in 15 ml polyethylene tubes containing the enzyme digestion mixture described below. Using collagenase D (1 mg/mL; Sigma-Aldrich) and DNase I (20 U/mL; Sigma-Aldrich), gingival tissues were minced and digested for 45 min at 37°C on a shaker. Next, tissues were passed through a 70-mm cell strainer, washed twice in complete RPMI 1640 medium (Gibco, Cat# 11875119), and centrifuged at 500g for 3 minutes at room temperature. Procedure details are provided in the supplementary materials.

2.5 Flow cytometry

Procedure details for flow cytometry are listed below according to previous protocol with minor modifications (Jiang, Wang, et al. 2022).

1. Prepare cells for flow cytometric staining using sodium azide-free buffers.
2. Wash cells one time in sodium azide- and protein-free Dulbecco's Phosphate Buffered Saline (1X DPBS).
3. Resuspend cells at 1×10^6 cells/ml in sodium azide- and protein-free 1× DPBS.
4. Add 1 µl of BD Fixable Viability Stain 620 Stock Solution for each 1 ml of cell suspension (1:1000) and vortex immediately.
5. Incubate the mixture for 10-15 minutes at room temperature or 2-8°C protected from light.
6. Wash cells twice with 2 ml of BD Pharmingen Stain Buffer (FBS).
7. Decant the supernatant and gently mix to disrupt the cell pellet.
8. Resuspend the cells in Stain Buffer (FBS) or equivalent.
9. Add 1 µl of BD Pharmingen CD 16/CD32 Stock Solution for each 1 ml of cell suspension (1:1000) and vortex immediately.
10. Incubate the mixture for 5 minutes at room temperature or 2-8°C protected from light.
11. Proceed with steps 6 and 7 again.
12. The surface markers were stained with anti-CD206, anti-F4/80, anti-CD11c, and anti-CCR2 for 30 min at 4°C in a total volume of 100 µL for 30 min at 4 °C

13. Proceed with steps 6 and 7 again.
14. Fixed the cells in 1% methanol-free formaldehyde for 30 min at 4 °C.
15. Proceed with steps 6 and 7 again.
16. Resuspended the cells in 1× permeabilization buffer and incubated them for 5 min at RT.
17. Cells were stained with anti-CCL2 in a total volume of 100 μL for 30 min at 4 ℃.
18. Proceed with steps 6 and 7 again.
19. We used a flow cytometer to evaluate all of the samples, and the results were processed in CytoFLEX software.

2.6 Histopathology

Procedure details for histopathology are listed below according to previous protocol with minor modifications.
In all of the timed steps below, the slide racks should be placed into separate staining dishes. The solutions should be changed frequently because there is some carryover as slide racks are moved through the containers. How often reagents must be changed depends on how many samples are stained. Room temperature should be used for all steps and solutions.
1. Place the glass slides that hold the paraffin sections in staining racks. Clear the paraffin from the samples in three changes of xylene for 2 min per change.
2. Hydrate the samples as follows.
   i. Transfer the slides through three changes of 100% ethanol for 2 min per change.
   ii. Transfer to 95% ethanol for 2 min.
   iii. Transfer to 70% ethanol for 2 min.
   iv. Rinse the slides in running tap water at room temperature for at least 2 min.
3. Stain the samples in hematoxylin solution for 3 min.
4. Place the slides under running tap water at room temperature for at least 5 min.
5. Stain the samples in working eosin Y solution for 2 min.
6. Dehydrate the samples as follows.
   i. Dip the slides in 95% ethanol about 20 times.
   ii. Transfer to 95% ethanol for 2 min.
   iii. Transfer through two changes of 100% ethanol for 2 min per change.
7. Clear the samples in three changes of xylene for 2 min per change.
8. Place a drop of Permount over the tissue on each slide and add a coverslip. View the slides using a microscope.
Stain intensity will vary with species and fixative. The eosin stain will be more intense with alcohol-based fixatives than with strong cross-linker fixatives. Weak cross-linker fixatives tend to have poor uptake of hematoxylin stains.

3 RESULTS

Figures S1 and S2 below show supplementary H&E images for Figures 5B and 7C.

Figure legends

Figure S1. Supplementary diagram of H&E images in figure 5B. H&E staining of WT and $Sting^{Gt}$ periodontitis mice. The images shown are 14 dpi for all groups (scale bar, 200 μm). Each image is representative of a group of 4 mice at 14 dpi.

Figure S2. Supplementary diagram of H&E images in figure 7C. H&E staining showed hemi-maxillae histopathological changes after treatment with small molecule modulators (scale bar, 200 μm). Each image is representative of a group of 4 mice at 14 dpi.

Tables

Table S1

Tools required for ligature-induced periodontitis in mice.

Tool names	Purpose
6-0 Silk suture	To be tied around second molar, to facilitate bacterial accumulation and inflammation
needle-holding spring forceps	Passing suture through interdentium
Suture-tying forceps	Tying suture
Optical Scissors	Cutting suture
